# Epidemic trend, genetic characteristics, and transmission networks of HIV-1 among treatment-naive men who have sex with men in Hebei province, China

**DOI:** 10.3389/fmicb.2024.1405565

**Published:** 2024-08-08

**Authors:** Xinli Lu, Yingying Wang, Lin Ma, Meng Liu, Yan Li, Ning An, Xinyu Zhang, Xiangyun Tang, Qi Li

**Affiliations:** Department of AIDS Research, Hebei Key Laboratory of Pathogen and Epidemiology of Infectious Disease, Hebei Provincial Center for Disease Control and Prevention, Shijiazhuang, Hebei, China

**Keywords:** HIV-1, integrase strand transfer inhibitors, subtype, pretreatment drug resistance, network, MSM

## Abstract

**Introduction:**

Homosexual transmission has contributed greatly to the current HIV-1 epidemic in Hebei province, China. Dolutegravir (DTG) will be conditionally used as a component of free antiretroviral therapy (ART) according to manual for national free anti-AIDS treatment drugs (2023 edition) issued by China in June 2023. However, current genetic characteristics and pretreatment drug resistance (PDR) to proteinase inhibitors (PIs), reverse transcriptase inhibitors (RTs) and integrase strand transfer inhibitors (INSTIs) of HIV-1 in this population have remained unclear.

**Methods:**

Serial consecutive cross-sectional analyses for HIV- 1 infection trend, genetic characteristics, PDR and molecular transmission networks were conducted from 2018 to 2022. All of participants were HIV-1- infected MSM newly diagnosed at the HIV surveillance points (HSPs) in Hebei, China. Evidence of PDR was confirmed using the world health organization (WHO) list for surveillance of drug resistance mutations.

**Results:**

In this study, a total of 14 HIV-1 subtypes were circulating in the HSPs of Hebei province, China. CRF01_ AE (51.9%, 350/675), CRF07_BC (30.4%, 205/675), B (6.2%, 42/675) and URFs (5.8%, 39/675) were the four most predominant subtypes among MSM. And, CRF07_BC (*r* > 0) and URFs (*r* > 0) indicated an increasing trend, respectively; however, CRF01_AE (*r* < 0) showed a decline trend. The overall prevalence of HIV-1 PDR showed a substantial increase from 6.3% in 2018 to 7.9% in 2022. The prevalence of NNRTI-PDR was the highest (5.8%, 39/675), followed by INSTIs (2.4%, 16/675), NRTIs (0.6%, 4/675) and PIs (0.3%, 2/675). Furthermore, extensive HIV-1 strains bearing PDR were circulating in the MSM population via molecular transmission networks for major HIV-1 subtypes, especially CRF01_AE and CRF07_BC.

**Discussion:**

Our findings reflect that HIV-1 epidemic in the MSM population is complex and severe in Hebei, China. Therefore, it is urgent for us to implement more effective intervention measures to limit the further dissemination of HIV-1, especially the spread of HIV-1 INSTI-PDR strains.

## Introduction

In the past forty-three years, acquired immune deficiency syndrome (AIDS) caused by human immunodeficiency virus (HIV) has contributed to serious public health problem, public security, and the recession of economic and culture worldwide (Liu and Yuan, 2003). In developing countries, AIDS is still one of three global threats such as drinking water shortage, reduction of agricultural acreage and AIDS in the 21st century, threatening the survival of mankind. In 2022, the Joint United Nations Program on HIV and AIDS (UNAIDS) reported that 39 million people worldwide were living with HIV, 1.3 million people were newly infected with HIV and 630,000 died of AIDS-related illnesses (UNAIDS, 2023). HIV-1 of two types (HIV-1 and HIV-2) is the most frequent strain which resulted in the rapid spread of AIDS nationwide. In order to achieve the goal of ending AIDS epidemic by 2030, global governments and scientists have taken many measures to control and prevent HIV epidemic since the first identification of AIDS in America, such as the development of diagnosis methods, three 95-95-95 targets (i.e., 95% of the people living with HIV know their status; 95% of diagnosed people on antiretroviral therapy (ART) and 95% of people receiving ART virally suppressed), the surveillance of HIV diversity, vaccine research and so on.

In order to reduce recent HIV infection and death related to AIDS, China has taken effective measures such as joint interventions, ART, and scale-up of diagnostic testing to control HIV epidemic, and made big progress (Han et al., 2021; Hao et al., 2022; Zhao et al., 2023). HIV infections through blood transfusions and blood products have been basically blocked (0.0% in 2020 to 2022), and injecting drug transmission (0.4% in 2022) and mother-to-child transmission (0.2% in 2022) have been effectively controlled (Han et al., 2021; Han, 2023). AIDS treatment coverage and treatment success rate have reached more than 90% (Zhao et al., 2023). However, sexual contact transmission has been the most common infection route, accounting for more than 95%, and HIV-1 has spread out of its original risk group into general population via sexual transmission. By the end of 2022, there were 1.223 million people living with HIV (PLWH) in China (Han, 2023). Although the situation of HIV epidemic remains at a low prevalence level in China, HIV/AIDS prevention and treatment in China are still facing the arduous task and many questions, such as complex HIV diversity, drug resistance mutations (DRMs) and so on, which may take great challenges to the development of HIV-1 diagnostic testing, viral load measurements, vaccine development, and ART (Joris et al., 2020). Similar to Chinese HIV epidemic situation, Hebei faces the same questions. Different from heterosexuals (72.0%) in China, homosexuals have become the most frequent transmission route of HIV-1 in Hebei, for example, the proportion (more than 65.0%) of men who have sex with man (MSM) among newly diagnosed cases in Hebei was significantly higher than national level (25.6%) in 2022 alone (Lu et al., 2018; Joris et al., 2020; Wang et al., 2021).

Due to the high mutation, replication rates, double infection and super-infection of HIV, HIV recombinant forms are being increasingly complex (Arshan et al., 2021). The Los Alamos HIV Sequence Database^1^ indicated that up to now, at least 158 circulating recombinant forms (CRFs) have been confirmed globally, including 157 HIV-1 CRFs and one HIV-2 CRF. And, a large number of unique recombinant forms (URFs) are continually formed each year. Of 157 CRFs, more than 50 CRFs were identified in China, and they mainly distributed in the sexual contact population especially MSM (>40%). Particularly, the number of novel CRFs derived from Chinese sequences are rising rapidly after 2018, and three novel CRFs were confirmed among MSM in Hebei. Even, some CRFs recently identified contained drug resistance to anti-retroviral drugs (ARDs).

Since the initiation of Chinese “Four Frees and One Care” policy in 2004, 92.8% of PLWH have been initiating and receiving ART, and 97.0% have obtained the suppressed viral load ≦ 1,000 copies/mL as of 2022 (Zhao et al., 2023). However, approximately 3.0% of PLWH experienced ART failure, and HIV DRM was the main factor of ART failure. Even, HIV-1 low-level viraemia (50–1,000 copies/mL) has been the risk factor of ART failure, DRM and death (Lucas et al., 2018), accounting for approximately 5.0% of PLWH receiving ART. The prevalence of HIV DR corresponding to reverse transcriptase inhibitors (RTIs)-based first-line ART was higher than others among PLWH receiving ART (WHO, 2021). 51.2% of 28,510 patients experiencing ART failure harbored HIV-1 DRM in 2021: the DR prevalence of nucleoside reverse transcriptase inhibitors (NRTIs), nonnucleoside reverse transcriptase inhibitors (NNRTIs) and lopinavi/ritonavir (LPV/r) was 27.4, 48.8 and 1.8%, respectively (Zhao et al., 2023). Among naïve HIV-infected individuals, the overall prevalence of DR indicated an upward trend from 2004 to 2022, with a slow elevation in annual percent change of 3.11% between 2004 and 2015, and a rapid increment in annual percent change of 11.17% between 2016 and 2022 (Liu et al., 2023). In Hebei, our previous study (Lu et al., 2017a) indicated that the prevalence of HIV-1 DR in patients experiencing ART failure and naïve ART was 51.9 and 55.9%, respectively. However, no systematic research on pretreatment DR (PDR) of integrase strand transfer inhibitors (INSTIs) in China has been reported. Therefore, there is growing concern that the increasing of HIV-1 PDR prevalence could decline the efficacy of ART, representing a threat to ending AIDS epidemic by 2030.

In this study, we analyzed HIV-1 near full-length *pol* sequences (NFLPs) of MSM newly diagnosed as HIV-1 infection at the HIV surveillance points (HSPs) in Hebei of China between 2018 and 2022 and the near full-length genomes sequences (NFLGs) of URFs. Based on these data, we evaluated (1) the recent prevalence and recombinant patterns of HIV-1 from 2018 to 2022 in Hebei, China; (2) HIV-1 PDR to proteinase inhibitors (PIs), NRTIs, NNRTIs and INSTIs, and (3) HIV-1 molecular transmission network.

## Materials and methods

### Study design

Serial consecutive cross-sectional analyses for HIV-1 infection trend, genetic characteristics, HIV-1 PDR and molecular transmission networks were conducted from 2018 to 2022 in Hebei, China.

### Participants

All of participants were MSM newly diagnosed as HIV-1 antibody positive at the HSPs in Hebei, China. A total of 758 MSM were enrolled in this study between 2018 and 2022, including 151 in 2018, 165 in 2019, 169 in 2020, 124 in 2021 and 149 in 2022. They were ART-naïve adolescents and adults. The participants should meet the below criteria: (a) Have homosexual contacts; (b) their ages are ≥15 years. We obtained written informed consent from the participants, and collected their blood samples and baseline information via face-to-face interviews in a private room at the HSPs of 11 municipal Centers for Disease Control and Prevention of Hebei province, China.

### HIV-1 laboratory tests

#### HIV-1 antibody, CD4 cell counts, and recent infection testing

5 mL whole blood sample was collected from each participant. 50 μL whole blood sample was used to test CD4 cell counts using a flow cytometry (Becton–Dickinson, Franklin Lakes, NJ, USA). For the testing related to HIV-1, blood plasma was separated from the remaining whole blood by centrifuging with a rotate speed of 3,000 rpm for five minutes. All participants were confirmed as HIV-1 infection by Western blot test (WB; MP Biomedical Asia Pacific Pte. Ltd., Singapore) between 2018 and 2022, respectively.

Recent HIV-1 infection was tested using HIV-1 LAg-Avidity EIA except in the below conditions: (1) CD4 cell counts<200 cells/μL; (2) AIDS patients; (3) the known long-term HIV-1 infection. Recent HIV-1 infection status was determined according to the manufacturer’s instructions (HIV-1 LAg-Avidity EIA kit, Beijing jinhao pharmaceutical Co., LTD, China).

#### Viral RNA extraction, amplification, and subtypes identification

Viral RNA was extracted from 200 μL of blood plasma using the Roche MagNa pure total RNA kit (Qiagen, Valencia, CA, USA). Firstly, NFLP gene fragment (HXB2:2068–5,221) was amplified for HIV-1 subtypes identification using reverse transcription-polymerase chain reaction (RT-PCR) and nested PCR. RT-PCR was operated using M-MLV 4 One-Step RT-PCR kit (Beijing Biomed Gene Technology Co., Ltd., Beijing, China) according to the manufacturer’s instructions, and using the below primers: Pol-1e-1R: 5′-TGGAAATGTGGRAARGARGGAC -3′ (forward) and Pol-x-1R: 5′- CCTGTATGCARMCCCCAATATGTT −3′ (reversed). Nested PCR was operated using TaKaRa Premix Taq (TaKaRa Biotechnology, Dalian, China) using the product of RT-PCR as a template. The primers of nested PCR were as follows: Pol-3-2R: 5′- ACTGAGAGACAGGCTAATTTTTTAGGGA −3′ (forward) and Pol-4e-2R: 5′- CTCCTAGTGGGATRTGTACTTCTGARCTTA −3′ (reversed). As the previous cycling conditions reported by us (Lu et al., 2017a), RT-PCR and nested PCR were performed. The PCR positive products were sequenced using Sanger’s method by Beijing Biomed Gene Technology Co., Ltd. (Beijing, China).

Raw original reaction sequences sequenced by gene-sequencing company were assembled, aligned and manual editing using Contig Express 9.1 and Bio-Edit 7.0 software, respectively. HIV-1 subtypes were preliminarily inferred using the online HIV Blast Microsoft,^2^ followed by a neighbor-joining (N-J) phylogenetic tree based on NFLP gene sequences using MEGA 7.0 with 1,000 bootstrap replicates. The reference sequences (A–D, F–H, J, K, O, CRF01_AE, CRFs_0107, CRFs_01C, CRFs_01B and others) were downloaded from the HIV database.^3^ And, subtypes were confirmed using the online REGA HIV-1 Subtyping Tool-Version 3.0.^4^ For novel HIV-1 URFs based on the NFLPs, their NFLGs were further amplified and sequenced as previous method described by us (Yang et al., 2022). Their recombinant patterns were identified by the recombinant analysis of online jpHMM, online RIP 3.0 and simplot 3.5.1.

#### HIV-1 DR and genetic network analysis

HIV-1 NFLPs obtained from participants were submitted to HIV DR databases.^5^ Gene mutations resistant to PIs, NRTIs, NNRTIs and INSTIs were analyzed using HIVDB algorithm version 9.5.1 according to the world health organization (WHO) -recommended criteria for PDR. Moreover, DR level was classified as four categories, including potential low-, low-, intermediate-, and high-level resistance for different ARDs.

HIV-1 molecular transmission networks were constructed using the NFLPs data sources in Hebei province, China. Real-time transmission network analyses were performed using HYPHY2.2.4 and Cytoscape v3.8.0. Pairwise genetic distances were calculated using the Tamura-Nei 93 (TN93) model. A genetic distance threshold of 0.015 substitutions/site was selected to construct networks because this threshold is consistent with recent and rapid transmission. Molecular transmission networks were visualized using Cytoscape v3.8.0.

#### Statistical analysis

Statistical analysis was conducted using SPSS 23.0 (SPSS Inc. Chicago, IL, USA). Means or frequencies were used to summarize demographic data. Differences and the epidemic trend in categorical variables were analyzed using the chi-square (χ^2^) test and χ^2^-trend, respectively. The pairwise test of independent variable in year and city was carried out using bonferroini method. All tests were two-sided *p* values and *p*-values <0.05 were considered statistically significant. Epidemic trend of HIV-1 subtypes with a *p*-value of 0.05 (χ^2^-trend) was analyzed using Spearman method: *r* < 0 denotes a negative correlation, and *r* > 0 denotes a positive correlation.

## Results

### Study population

**Figure 1:**
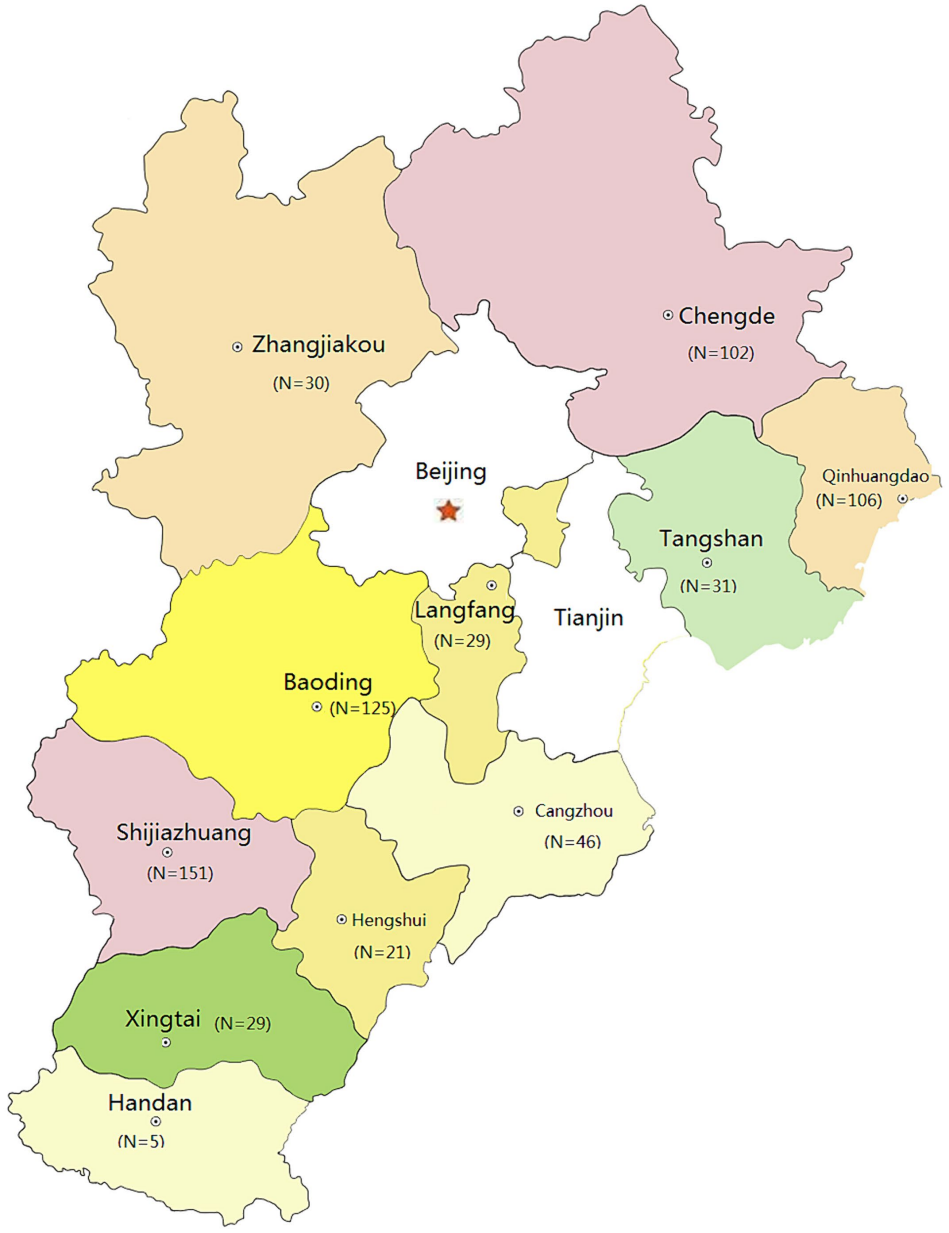
Geographic distribution of participants obtained from the HIV surveillance points in eleven cities of Hebei province, China. This figure was adapted from open access map: http://map.ps123.net/china/14.html with Microsoft PowerPoint 2016. In this figure, eleven areas denote all cities of Hebei province, including Zhangjiakou, Chengde, Qinhuangdao, etc. *N* denotes the number of participants.

As presented in Table 1, a total of 675 NFLPs were generated from 758 participants` plasma samples and used in this analysis, achieving a PCR successful rate of 89.1%. These data were collected between 2018 and 2022 from all 11 cities of Hebei province (Figure 1), China. Among 675 participants, the Han ethnicity accounted for 93.0%. The most frequent age strata were 25–49 years (61.9%, 418/675), followed by 15–24 years (21.9%, 148/675), and ≥ 50 years (16.1%, 109/675). The most frequent marital status was unmarried (48.6%, 328/675), followed by married (37.2%, 251/675), and divorced/widowed (14.2%, 96/675). CD4 cell counts >200 cells/μL accounted for 87.7% (592/675). 18.7% (126/675) had ever been infected with sexually transmitted disease (STD). Recent HIV-1 infections accounted for 25.9% (175/675). Each participant had at least one homosexual contact partner. 64.3% of 675 participants had 2 to 9 homosexual partners. For sample sources, 63.4% (428/675) were voluntary consultation and testing (VCT). Their occupations were grouped into five categories: farmer was the most common (25.8%, 174/675), followed by service (24.1%, 163/675), housework/freelance (23.4%, 158/675), cadre/staff/teacher (17.3%, 117/675), and student (9.3%, 63/675). 35.4% (239/675) of participants had the educational level of college or above.

**Table 1:** Demographic characteristics of HIV-infected subjects in sentinel surveillance surveys in Hebei, 2018–2022.

Variable	Total	2018		2019		2020		2021		2022		Chi square		Chi square for trend	
		Cases	Pol	Cases	Pol	Cases	Pol	Cases	Pol	Cases	Pol	χ^2^	*p*	χ^2^-trend	*p*
Gender															
Male	758 (675)	151	128	165	147	169	152	124	109	149	139				
Age												28.527	<0.001	12.346	<0.001
15–24	164 (148)	29	26	46	40	42	37	26	25	21	20			2.734	0.098
25–49	476 (418)	111	92	99	90	95	86	79	66	92	84			2.544	0.111
≥50	118 (109)	11	10	20	17	32	29	19	18	36	35			16.034	<0.001
Occupation												49.600	<0.001	8.008	0.005
Service	178 (163)	45	40	41	37	37	32	26	25	29	29			4.9	0.026
Student	67 (63)	15	13	18	18	13	13	9	8	12	11			1.013	0.314
Housework/freelance	178 (158)	33	30	33	29	38	33	50	43	24	23			0.342	0.559
Cadre/staff/teacher	131 (117)	22	19	39	34	25	22	19	17	26	25			0.084	0.772
Farmer	204 (174)	36	26	34	29	56	52	20	16	58	51			6.058	0.014
Marital status												20.975	0.007	0.100	0.752
Divorced/widowed	112 (96)	19	17	24	22	27	21	22	19	20	17			0.236	0.627
Unmarried	359 (328)	80	71	88	78	57	53	63	57	71	69			1.268	0.260
Married	287 (251)	52	40	53	47	85	78	39	33	58	53			0.646	0.422
Nationality												1.405	0.843	0.438	0.508
Han	702 (628)	139	116	150	134	159	149	115	100	139	129				
Minority	56 (47)	12	12	15	13	10	3	9	9	10	10				
Educational level												23.929	0.002	0.315	0.575
Junior school or below	296 (267)	53	48	61	53	77	72	47	41	58	53			0.520	0.471
High school	194 (169)	38	31	37	33	58	50	29	24	32	31			0.254	0.614
College or above	268 (239)	60	49	67	61	34	30	48	44	59	55			0.076	0.783
Initial CD4 counts (cells/μL)											161.008	<0.001	69.729	<0.001	
≤200	93 (83)	0	0	1	0	53	48	6	5	33	30			36.552	<0.001
201–500	413 (372)	71	60	86	79	88	79	79	70	89	84			7.760	0.005
>500	252 (220)	80	68	78	68	28	25	39	34	27	25			51.198	<0.001
STD history												0.463	0.977	0.234	0.628
Yes	133 (126)	29	27	29	26	28	28	22	21	25	24				
No	625 (549)	122	101	136	121	141	124	102	88	124	115				
Homosexual partner												62.485	<0.001	3.601	0.058
1	158 (137)	29	23	26	24	49	41	14	11	40	38			1.529	0.216
2–9	494 (434)	104	88	129	113	109	100	78	67	74	66			19.746	<0.001
≥10	106 (104)	18	17	10	10	11	11	32	31	35	35			21.676	<0.001
Sample source												72.988	<0.001	0.411	0.521
VCT	470 (428)	102	83	120	117	82	75	79	71	89	82			4.528	0.033
Visit test	163 (143)	20	18	22	18	62	56	17	12	42	39			9.606	0.002
Volunteer blood donor	26 (22)	6	6	1	1	14	11	3	2	2	2			0.389	0.533
Special survey	97 (91)	23	21	22	20	11	10	25	24	16	16			0.149	0.700
Infection status												4.608	0.330	3.281	0.070
LT	559 (500)	117	99	131	116	125	115	87	77	107	101				
RI	199 (175)	34	29	34	31	44	37	37	32	42	38				

LT, Long-term infection; RI, Recent infection; VCT, voluntary consultation and testing; STD, sexually transmitted disease.

During the five-year period, significant differences in the HIV-1 infections were found by age (*p* < 0.001), occupation (*p* < 0.001), marital status (*p* = 0.007), educational level (*p* = 0.002), initial CD4 counts (*p* < 0.001), homosexual partner (*p* < 0.001), and sample source (*p* < 0.001). Furthermore, increasing trends were observed in the proportion of participants aged >50 (*p* < 0.001), being service (*p* = 0.026) and farmer (*p* = 0.014), initial CD4 counts (*p* < 0.001), homosexual partners with 2–9 (*p* < 0.001) and ≥ 10 (*p* < 0.001), VCT (*p* = 0.033) and visit test (*p* = 0.002) within sample source group.

### HIV-1 genotyping

As shown in Table 2, the HIV-1 epidemic in Hebei province was driven by 14 kinds of HIV-1 subtypes according to the overall analysis of HIV-1 NFLP and NFLG gene sequences, including 10 CRFs, three simple subtypes and a proportion of URFs. CRF01_AE was the most common subtype, accounting for 51.9% (350/675), followed by CRF07_BC (30.4%, 205/675), B (6.2%, 42/675), URFs (5.8%, 39/675), and CRF55_01B (2.2%, 15/675). The above five subtypes were annually circulating in Hebei between 2018 and 2022, and recent HIV-1 infections were mainly distributed in these subtypes. Furthermore, the distribution of major subtypes showed no differences (χ^2^ = 6.481, *p* = 0.166) between long-term infection and recent infection. Other subtypes indicated small-scale sporadic characteristics, and had a prevalence of ≤1.0%. The number of HIV-1 subtypes among MSM at the HSPs showed a significant increase from 6 in 2018 to 10 subtypes in 2022. And, new subtypes occurred each year (Table 2).

**Table 2:** Distribution of HIV-1 subtypes and drug resistance among MSM at HIV surveillance points in Hebei, 2018–2022.

Subtype	2018 (*N* = 128)		2019 (*N* = 147)		2020 (*N* = 152)		2021 (*N* = 109)		2022 (*N* = 139)		Infection status		Total
	Cases	DR	Cases	DR	Cases	DR	Cases	DR	Cases	DR	LT	RI	
CRF01_AE	82 (64.1)	3 (5.5)	77 (52.4)	7 (5.4)	83 (54.6)	10 (9.2)	41 (37.6)	3 (2.8)	67 (48.2)	5 (3.6)	246 (36.4)	104 (15.4)	350 (51.9)
CRF07_BC	26 (20.3)	1 (0.8)	51 (34.7)	7 (4.8)	45 (29.6)	7 (4.6)	38 (34.9)	6 (5.5)	45 (32.4)	4 (2.9)	155 (23.0)	50 (7.4)	205 (30.4)
B	14 (10.9)	3 (2.3)	9 (6.1)	-	6 (4.0)	-	6 (5.5)	-	7 (5.0)	-	35 (5.2)	7 (1.0)	42 (6.2)
URFs	2 (1.6)	-	2 (1.4)	-	10 (6.6)	-	15 (13.8)	-	10 (7.2)	-	31 (4.6)	8 (1.2)	39 (5.8)
CRF55_01B	3 (2.3)	1 (0.8)	4 (2.7)	-	3 (2.0)	-	2 (1.8)	-	3 (2.2)	1 (0.7)	13 (1.9)	2 (0.3)	15 (2.2)
C	1 (0.8)	-	-	-	-	-	-	-	-	-	-	1 (0.1)	1 (0.1)
CRF80_0107	-	-	3 (2.0)	-	1 (0.7)	-	1 (0.9)	-	-	-	4 (0.6)	1 (0.1)	5 (0.7)
CRF68_01B	-	-	-	-	2 (1.3)	-	1 (0.9)	-	1 (0.7)	1 (0.7)	3 (0.4)	1 (0.1)	4 (0.6)
CRF65_CPX	-	-	1 (0.7)	-	1 (0.7)	1 (0.6)	2 (1.8)	-	3 (2.2)	-	7 (1.0)	-	7 (1.0)
CRF08_BC	-	-	-	-	1 (0.7)	-	2 (1.8)	-	-	-	3 (0.4)	-	3 (0.4)
CRF103_01B	-	-	-	-	-	-	1 (0.9)	-	-	-	1 (0.1)	-	1 (0.1)
CRF113_0107	-	-	-	-	-	-	-	-	1 (0.7)	-	-	1 (0.1)	1 (0.1)
A1	-	-	-	-	-	-	-	-	1 (0.7)	-	1 (0.1)	-	1 (0.1)
CRF 112_01B	-	-	-	-	-	-	-	-	1 (0.7)	-	1 (0.1)	-	1 (0.1)
HIV-1 PDR													
PIs	-	-	-	-	2 (1.3)	ATV/r (I); LPV/r (L); DRV/r (L)	-	-	-	-	2 (0.3)	-	2 (0.3)
NRTIs	-	-	1 (0.7)	AZT (L)	2 (1.3)	ABC/TDF (H);AZT (L); FTC/3TC (H)	1 (0.9)	AZT (L)	-	-	4 (0.6)	-	4 (0.6)
NNRTIs	5 (3.9)	EFV/ETR/RPV (I); NVP (H)	9 (6.1)	DOR/EFV/NVP/RPV (H); ETR (L)	11 (7.2)	ETR (I),DOR/EFV/NVP/RPV (H)	7 (6.4)	ETR (I);DOR/EFV/NVP/RPV (H)	7 (5.0)^a,b^	ETR/RPV (L); EFV/NVP (H)	31 (0.6)	9 (1.3)	40 (5.9)
INSTIs	3 (2.3)	EVG/RAL(L);BIC/CAB/DTG (L)	4 (2.7)	EVG (H);BIC/CAB/DTG (L)	3 (2.0)	BIC/DTG (L);CAB (I);EVG/RAL (H)	1 (0.9)	EVG/RAL (L)	5 (3.6)^b^	EVG (H;) RAL (L)BIC/CAB/DTG (L)	12 (1.8)	4 (0.6)	16 (2.4)

"-" denotes " none "; INSTIs, integrase strand-transfer inhibitors; H, high-level resistance; L, low-level resistance; I, intermediate-level resistance; PL, potential low-level resistance; LT, Long-term infection; RI, Recent infection; PIs, proteinase inhibitor; NRTIs, nucleoside reverse transcriptase inhibitors; NNRTIs, non nucleoside reverse transcriptase inhibitors; ^a^One participant carried three mutations (S68SG, L74M and A98G) in 2022, but both S68SG and L74M can`t cause drug resistance; ^b^one participant carried two mutations (K103N and S153F) in 2022.

**Figure 2:**
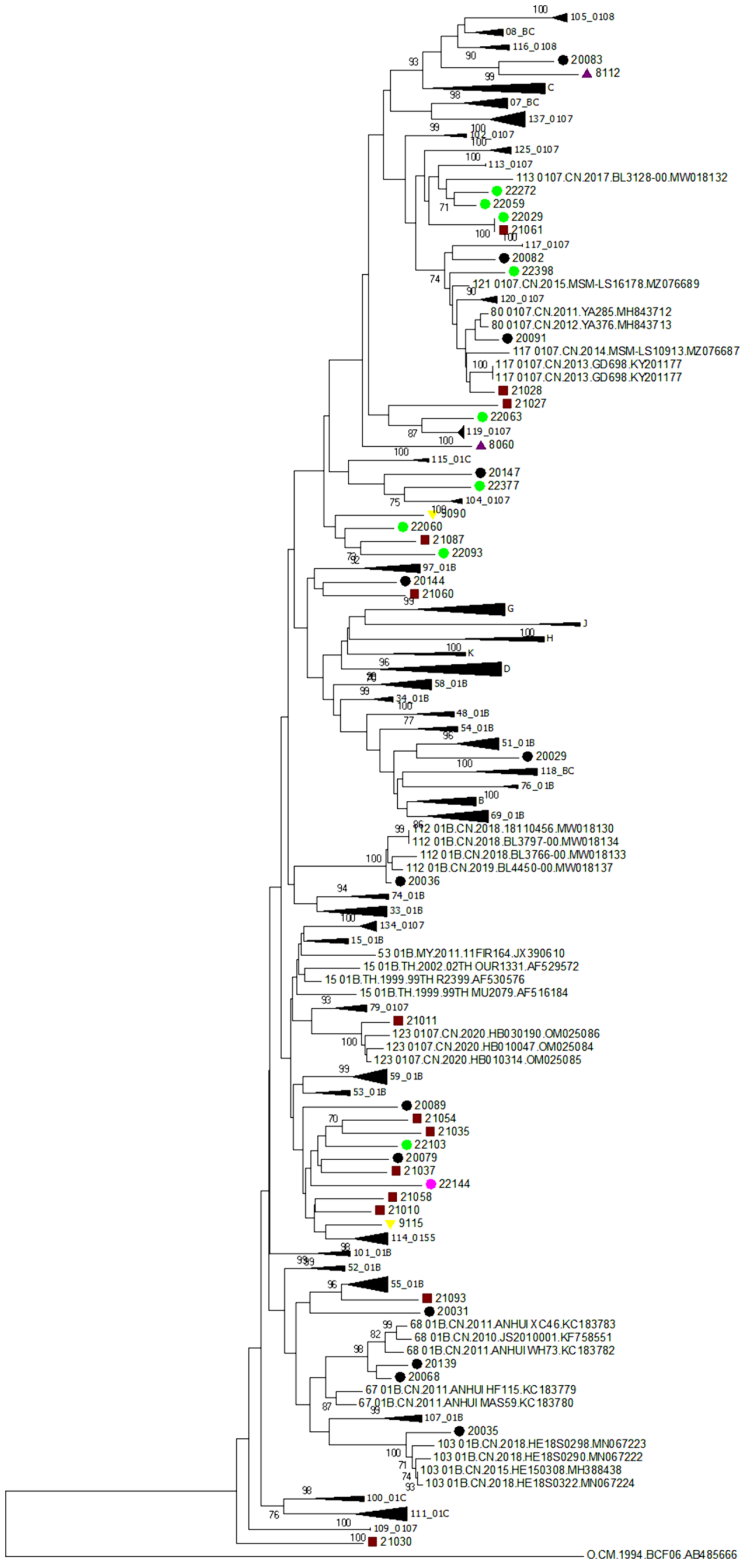
Phylogenetic tree based on HIV-1 near full-length pol gene sequences. The neighbor-joining tree was constructed based on HIV-1 near full-length *pol* sequences (NFLPs) identified in this study using MEGA 7.0. This figure identified all HIV-1 URFs which are based on NFLPs. Bootstrap values ≥70% are shown in the tree. The scale length indicates 2% nucleotide sequence divergence. Purple triangle, yellow inverted triangle, black dots, red square and pink dot denote participants identified in 2018, 2019, 2020, 2021, and 2022, respectively.

**Figure 3:**
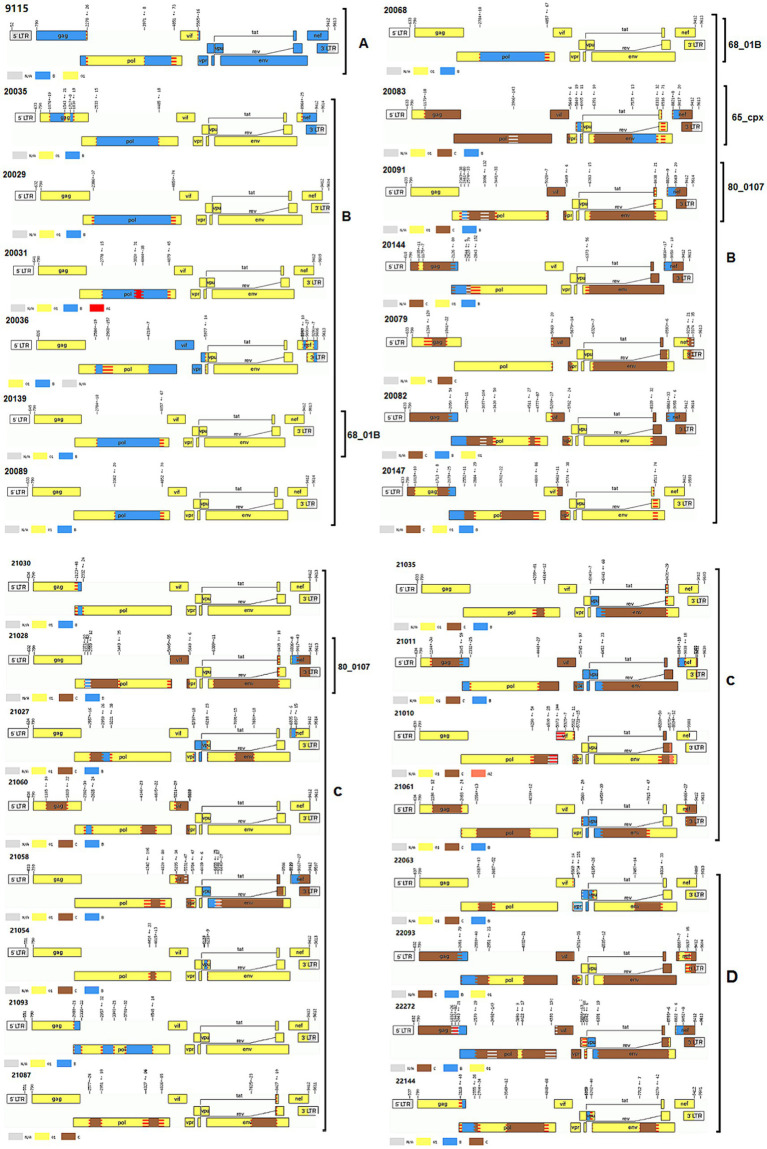
Recombinant patterns of URFs based on HIV-1 near full-length genomes. The gene mosaic maps of HIV-1 near full-length genomes were analyzed using the online jpHMM (http://jphmm.gobics.de/). A, B, C and D denote the gene mosaic maps of novel recombinant forms found in 2019, 2020, 2021, and 2022, respectively. The number such as 9,115, 20,035 and so on denote sample ID. Other numbers listed in each gene map are gene recombinant breakpoints places.

Table 3 showed that CRF07_BC (*r* = 0.083, *p* = 0.034) and URFs (*r* = 0.145, *p* = 0.000) indicated an increasing trend, respectively; however, CRF01_AE showed a decline trend (*r* = −0.110, *p* = 0.005). Particularly, the proportion of URFs indicated an obvious increasing trend from 1.6% in 2018 to 7.2% in 2022. As presented in Figure 2 and Table 4, 44 NFLP-URFs were identified between 2018 and 2022. 30 NFLGs from participants with 44 NFLP-URFs were successfully sequenced in order to confirm their recombinant forms. The recombinant structure analysis (Table 4; Figure 3) indicated that 3 of 30 NFLGs were identified as CRFs, including two CRF68_01Bs, two CRF80_0107s and one CRF65_cpx. The remaining 25 NFLG-URFs and 14 NFLP-URFs were confirmed as the real URFs in this study.

**Table 3:** Epidemic trend of main HIV-1 subtypes circulating at the HSPs in Hebei province, China.

Subtype	2018	2019	2020	2021	2022	Chi square		Chi square for trend		Spearman	
						χ^2^	*p*	χ^2^-trend	*p*	*r*	*p*
CRF01_AE	82	77	83	41	67	14.427	0.006	7.640	0.006	-0.110	0.005
CRF07_BC	26	51	45	38	45	10.448	0.035	4.371	0.037	0.083	0.034
B	14	9	6	6	7	6.116	0.191	2.909	0.088	-0.069	0.078
URFs	2	2	10	15	10	24.269	0.000	13.193	0.000	0.145	0.000
CRF55_01B	3	4	3	2	3	0.256	0.992	0.057	0.811	0.010	0.802
Total	127	143	147	102	132						

First, the difference between different subtypes is analyzed using Chi square (χ^2^) test. Further analysis will be carried out using Chi square for trend when a *p*-value (χ^2^-test) for HIV-1 subtype is less than 0.05. Lastly, we use Spearman method to analyze epidemic trend of HIV-1 subtypes with a *p*-value of 0.05 (χ^2^-trend): *r* < 0 denotes a negative correlation, and *r* > 0 denotes a positive correlation. *p*-values < 0.05 were considered statistically significant.

**Table 4:** Recombinant patterns of URFs based on HIV-1 near full-length *pol* genes and near full-length genomes.

Year	Near full-length *pol* gene		Near full-length genome	
	Pattern	Cases	Pattern	Cases
2018	CRF01_AE/C	1	0	0
	CRF01_AE/B/C	1	0	0
2019	CRF01_AE/B	2	CRF01_AE/B	1
2020	CRF01_AE/C	2	CRF01_AE/C	1
	CRF01_AE/B	6	CRF01_AE/B	6
	CRF01_AE/B/C	5	CRF01_AE/CRF07_BC	5
	CRF68_01B/A1	1	CRF68_01B/CRF01_AE	1
2021	CRF01_AE/C	9	CRF01_AE/C	2
			CRF01_AE/CRF07_BC	4
	CRF01_AE/B	2	CRF01_AE/B	2
	CRF01_AE/B/C	5	CRF01_AE/CRF07_BC	4
2022	CRF01_AE/B	3	CRF01_AE/B	0
	CRF01_AE/B/C	6	CRF01_AE/CRF07_BC	3
	CRF01_AE/C	1	CRF01_AE/CRF07_BC	1
Total		44		30

### Viral gene mutations resistant to PIs, NRTIs, NNRTIs, and INSTIs

As shown in Table 2, the overall prevalence of HIV-1 PDR among MSM showed a substantial increase from 6.3% (8/128) in 2018 to 11.8% (18/152) in 2020, while a sharp decrease to 7.9% (11/139) in 2022 was observed. In the five consecutive years, the PDR prevalence of NNRTIs was the highest (5.8%, 39/675), followed by INSTIs (2.4%, 16/675), NRTIs (0.6%, 4/675) and PIs (0.3%, 2/675). The prevalence of NRTI-, PI- and NNRTI-PDR reached the highest value in 2020, respectively, and then decreased obviously. Of these three ARDs, NNRTI-PDR kept a moderate level from 2018 to 2022. However, the INSTI-PDR prevalence indicated an increase from 2.3% (3/128) in 2018 to 2.7% (4/147) in 2019, while a sharp decrease to 0.9% (1/109) in 2021 was observed, and then increased significantly to 3.6% (5/139) in 2022. Additionally, 88.3% (53/60) of participants with PDR were infected with main subtypes such as CRF01_AE (46.7%, 28/60) and CRF07_BC (41.7%, 25/60).

### HIV-1 genetic transmission networks

**Figure 4:**
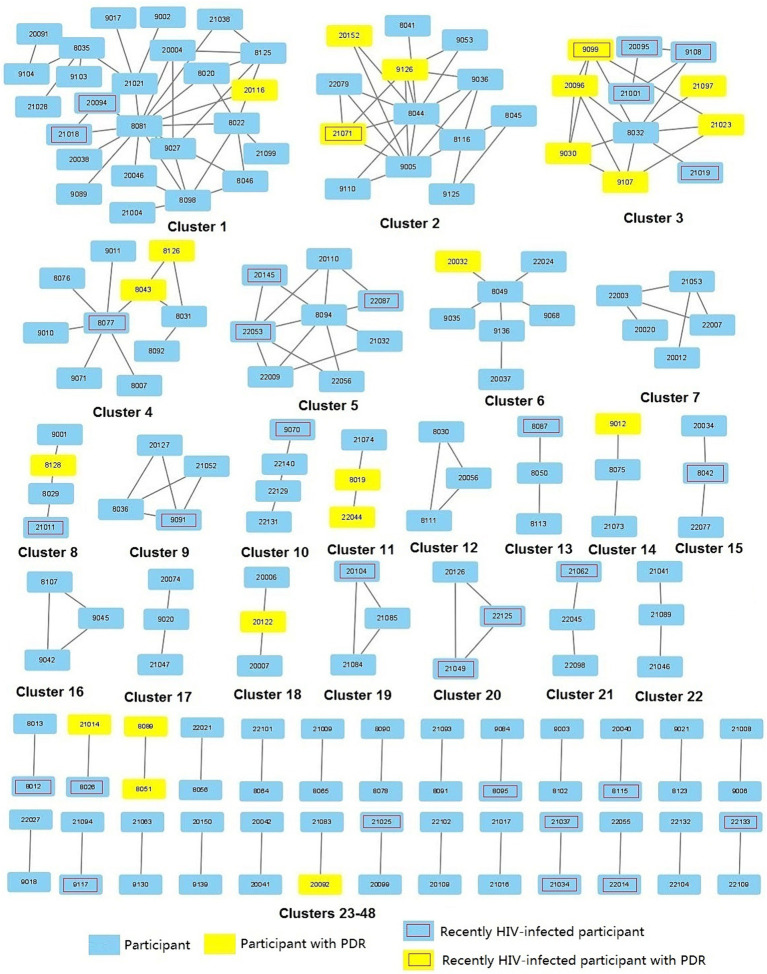
HIV-1 molecular transmission networks based on HIV-1 Near full-length pol gene sequences from all HIV-1 sequences identified in this study. There were 48 clusters in this study. There were seven large clusters containing 5 participants, including two CRF07_BC clusters (clusters 1 and 3), 5 CRF01_AE clusters (clusters 2, and 4–7). Other clusters contained 2–4 participants.

We used HIV-1 NFLPs data sources to characterize molecular transmission network in Hebei, China. In this study, a total of 675 HIV-1 NFLPs were used to infer HIV-1 molecular transmission networks. There were 179 of 675 NFLPs detected within the genetic transmission networks with a genetic distance threshold of 0.015, and they were distributed in 48 transmission clusters (Figure 4). These 48 transmission clusters included 25 (52.1%), 14 (29.2%), 6 (12.5%), 1 (2.1%), and 2 (4.2%) clusters for subtypes CRF01_AE, CRF07_BC, B, CRF65_cpx, and CRF55_01B, respectively. Within the networks, most of HIV-1 subtypes were CRF01_AE (51.4%, 92/179) and CRF07_BC (36.3%, 65/179).

Figure 4 indicated that seven of 48 clusters contained five or more participants, including five CRF01_AE clusters (clusters 2, 4–7) and two CRF_07 BC clusters (clusters 1 and 3). Other clusters contained 2–4 participants. Clusters containing HIV-1 PDR and recently HIV-1 infected participants accounted for 22.9% (11/48) and 43.8% (21/48), respectively. Five clusters containing PDR were found in seven major large clusters in this study, and not only PDR strains but also recently HIV-1 infections were circulating in four of five clusters with HIV-1 PDR. Table 5 showed that cluster 1 contained PI-mutations (F53L/I54L), cluster 2 contained INSTI-mutations (G163R and S153SF), cluster 3 contained NNRTI- (K103N and Y188L) and INSTI-mutations (Q148QH), cluster 4 contained INSTI-mutations (G163R), and cluster 6 contained PI-mutations (M46L).

**Table 5:** Variable characteristics of HIV-1 genetic transmission networks in Hebei province, 2018–2022.

Variable	Genetic networks								Number (%)		Chi square	
	Cluster 1 (*n* = 25)	Cluster 2 (*n* = 13)	Cluster 3 (*n* = 11)	Cluster 4 (*n* = 10)	Cluster 5 (*n* = 8)	Cluster 6 (*n* = 7)	Cluster 7 (*n* = 5)	Other clusters with 2–4 subjects^a^ (*n* = 100)	Clustering frequency *n* (%)	Total (*n* = 675)	χ^2^	*p*
Age											5.807	0.055
15–24	1	2	4	1	0	1	0	21	30 (20.3)	148		
25–49	18	11	7	9	8	6	5	60	124 (29.7)	418		
≥50	6	0	0	0	0	0	0	19	25 (22.9)	109		
Occupation											7.322	0.120
Service	3	2	3	1	2	1	1	23	36 (22.1)	163		
Student	0	2	2	0	0	3	0	10	17 (27.0)	63		
Housework/freelance	7	4	5	5	1	2	1	20	45 (28.5)	158		
Cadre/staff/teacher	8	2	0	2	5	1	1	16	35 (30.0)	117		
Farmer	7	3	1	2	0	0	2	31	46 (22.3)	174		
Marital status											4.192	0.123
Divorced/widowed	6	1	0	2	0	0	1	15	25 (26.0)	96		
Unmarried	7	9	11	7	8	4	3	49	98 (29.9)	328		
Married	12	3	0	1	0	3	1	36	56 (22.3)	251		
Nationality											1.468	0.226
Han	23	11	9	6	8	6	5	95	163 (26.0)	628		
Minority	2	2	2	4	0	1	0	5	16 (34.0)	47		
Educational level											5.051	0.080
Junior school or below	13	2	4	3	2	1	2	43	70 (26.2)	267		
High school	6	7	3	4	1	3	3	28	55 (32.5)	169		
College or above	6	4	4	3	5	3	0	29	54 (22.6)	239		
Initial CD4 counts (cells/μL)											4.704	0.030
200–500	18	7	6	6	6	4	5	57	109 (24.0)	455		
>500	7	6	5	4	2	3	0	43	70 (31.8)	220		
STD history											0.975	0.323
Yes	5	4	1	0	2	4	0	13	29 (23.0)	126		
No	20	9	10	10	6	3	5	87	150 (27.3)	549		
Homosexual partner											1.685	0.431
1	5	2	0	0	0	4	2	18	31 (22.6)	137		
2–9	16	10	8	8	4	3	2	66	117 (27.0)	434		
≥10	4	1	3	2	4	0	1	16	31 (29.8)	104		
Sample soure											3.158	0.368
VCT	15	10	7	9	7	5	4	51	108 (25.2)	428		
Visit test	6	1	3	0	1	2	1	20	34 (23.8)	143		
Volunteer blood donor	0	0	0	0	0	0	0	7	7 (31.8)	22		
Special survey	4	2	1	1	0	0	0	22	30 (33.0)	91		
Infection status											0.229	0.632
LT	22	11	5	9	4	6	4	74	135 (27.0)	500		
RI	3	2	6	1	4	1	1	26	44 (25.1)	175		
Drug resistance											1.610	0.204
Yes	2	3	6	2	0	1	0	9	23 (32.9)	70		
No	23	10	5	8	8	6	5	91	156 (25.8)	605		
Year within network											18.424	0.001
2018	7	4	1	7	1	1	0	27	48 (37.5)	128		
2019	6	6	4	3	0	3	0	15	37 (25.2)	147		
2020	6	1	2	0	2	2	2	16	31 (20.4)	152		
2021	6	1	4	0	1	0	1	24	37 (33.9)	109		
2022	0	1	0	0	4	1	2	18	26 (18.7)	139		
Cities within network											21.688	0.017
Qinhuangdao	8	2	11	2	1	1	0	13	38 (35.8)	106		
Baoding	4	3	0	6	1	0	0	24	38 (30.4)	125		
Shijiazhuang	8	0	0	0	6	0	3	26	43 (28.5)	151		
Cangzhou	2	0	0	0	0	0	2	3	7 (15.2)	46		
Chengde	2	8	0	2	0	5	0	19	36 (35.3)	102		
Tangshan	1	0	0	0	0	0	0	3	4 (12.9)	31		
Langfang	0	0	0	0	0	1	0	2	3 (10.3)	29		
Hengshui	0	0	0	0	0	0	0	4	4 (19.0)	21		
Zhangjiakou	0	0	0	0	0	0	0	6	6 (20.0)	30		
Xingtai	0	0	0	0	0	0	0	0	0 (0.0)	29		
Handan	0	0	0	0	0	0	0	0	0 (0.0)	5		
Subtype											3.042	0.551
CRF01_AE		√		√	√	√	√	√	92 (26.3)	350		
CRF07_BC	√		√					√	65 (31.7)	205		
B								√	15 (35.7)	42		
CRF55_01B								√	5 (33.3)	15		
CRF65_cpx								√	2 (28.6)	7		
PDR mutation in cluster^b^												
PIs	F53L/I54L					M46L		M46L				
NRTIs								T215TS				
NNRTIs			K103N; Y188L					E138EG/K; A98G; V179L; G190E				
INSTIs		G163R; S153SF	Q148QH	G163R								

LT, Long-term infection; RI, Recent infection; ^a^Other clusters includes 3 clusters containing four subjects (2 CRF01_AE clusters and 1 CRF07_BC cluster), 12 clusters containing three subjects (1 CRF55_01B cluster, 3 B clusters, 3 CRF07_BC clusters, and 5 CRF01_AE clusters), and 26 clusters containing 2 subjects (1 CRF65_cpx, 3 B clusters, 13 CRF01_AE clusters, 8 CRF07_BC clusters, and 1 CRF55_01B clusters). ^b^M46L can`t cause obvious resistance to PIs, and we don`t analyze it here. The pairwise test of independent variable by year and city was carried out using bonferroini method.

As shown in Table 5, participants were more likely to be within HIV-1 transmission clusters if aged 25–49 years, cadre/staff/teacher, unmarried, minority, high school or below, initial CD4 counts >500 cells/μL, no STD history, homosexual partners ≥10, special survey, long-term infection and drug resistance in general. In chi square analysis, clustering frequency in the MSM population was significantly related to CD4 counts (*p* = 0.030), year (*p* = 0.001) and city (*p* = 0.017). Clustering frequency had a significant difference among different calendar years, showing a substantial decrease from 37.5% in 2018 to 18.7% in 2022. Among eleven cities, there were significant clustering differences: Qinhuangdao had the highest clustering frequency (35.8%, 38/106), followed by Chengde (35.3%, 36/102), Baoding (30.4%, 38/125), Shijiazhuang (28.5%, 43/151). In initial CD4 counts, participants with >500 cells/μL had the higher clustering frequency (31.8%, 70/220) than those with 200–500 cells/μL (24.0%, 109/455). Among HIV-1 subtypes, clustering frequency was the highest in subtype B, accounting for 35.7% (15/42), followed by CRF55_01B (33.3%, 5/15), CRF07_BC (31.7%, 65/205), CRF65_cpx (28.6%, 2/7), and CRF01_AE (26.3%, 92/350). As the most frequent subtypes, although there was the lowest clustering frequency in CRF01_AE and the third largest clustering frequency in CRF07_BC, all of large clusters (cluster 1–7) were distributed in these two CRFs.

For seven main clusters, the transmission relationship of different subtypes within eleven cities of Hebei was generally diverse. Six (cluster 1–2, 4–7) of seven clusters with ≥5 participants (Table 5) were being in intercity links, with an apparent exception of cluster 3, such as a strong intracity link in Qinhuangdao for CRF07_BC. The strongest intercity transmission for CRF07_BC was identified in Qinhuangdao-Baoding-Shijiazhuang-Cangzhou-Chengde-Tangshan (cluster 1). The major intercity transmissions for CRF01_AE were also found in Qinhuangdao-Baoding-Chengde (cluster 2, 4), Qinhuangdao-Baoding-Shijiazhuang (cluster 5), Qinhuangdao-Chengde-Langfang (cluster 6) and Shijiazhuang-Cangzhou.

## Discussion

In order to monitor the prevalence of HIV and influencing factors, analyze HIV epidemic trend, and evaluate the changing of HIV plague in the special populations in different areas, China has set up numerous HSPs nationwide. For our current work, HIV-1 epidemic trend, PDR and molecular transmission networks among ART-naïve MSM were investigated using five consecutive cross-sectional data obtained from the HSPs in Hebei Province, China. The primary observation from our investigation showed that 14 kinds of HIV-1 subtypes were circulating among MSM newly diagnosed at the HSPs in Hebei, including 10 CRFs, three simple subtypes and a proportion of URFs. Of these subtype strains, the proportion of HIV-1 recombinants was 93.5% (631/675), which is in accordance with overall data (80 to 99%) in China (Joris et al., 2020). CRF01_AE (51.9%), CRF07_BC (30.4%), B (6.2%), and URFs (5.8%, 39/675) were the four dominant subtypes circulating in the MSM population in Hebei, which shares similarities with that in China (Ye et al., 2022). The study indicated that a total of 38 HIV-1 subtypes were identified in China (Ye et al., 2022). Of 38 subtypes, CRF01_AE (44.3%) was the most frequent subtype, followed by CRF07_BC (28.3%), B (13.9%) and URFs (5.9%) in the MSM population. However, the prevalence of HIV-1 subtypes in Hebei is significantly different from that in the western and southern provinces of China (Su et al., 2014; Zhang et al., 2022). Key data from our study illustrated that the prevalence of dominant HIV-1 subtypes had changed continually from 2018 to 2022 among MSM from the HSPs in Hebei: CRF07_BC (*r* = 0.083, *p* = 0.034) and URFs (*r* = 0.145, *p* = 0.000) indicated an increasing trend, respectively; however, CRF01_AE showed a decline trend (*r* = −0.110, *p* = 0.005). Furthermore, the distribution of these four major subtypes in long-term infection and recent infection was not different, and these four major subtypes were the source of recent HIV-1 infection. The number of HIV-1 subtypes among MSM at the HSPs showed a significant increase from 6 in 2018 to 10 subtypes in 2022, and new subtypes occurred each year. Our study has unearthed a crucial finding regarding URFs: the proportion of URFs presented a rapid increasing trend from 1.6% in 2018 to 7.2% in 2022, and 1.2% of URFs were the recent infection. Moreover, as the dominant subtypes circulating in Hebei, CRF01_AE, CRF07_BC and B formed the essential architectures of URFs. In Hebei, MSM has overtaken other transmission routes and become the most predominant transmission pathway of HIV-1. Therefore, the above changes in the province level HIV-1 diversity among MSM at the HSPs illustrate a shift in homosexual risk behavior patterns and reflect HIV-1 epidemic trend in Hebei.

In our study, we analyzed HIV-1 PDR to PIs, NRTIs, NNRTIs and INSTIs and their changes among ART-naive MSM in 2018–2022. Particularly, the systematic research on the province level INSTI-PDR in Hebei was first conducted and analyzed. Our study showed that the overall prevalence of HIV-1 PDR among MSM at the HSPs in Hebei presented a substantial increase from 6.3% in 2018 to 11.8% in 2020, while a sharp decrease to 7.9% in 2022 was observed, which corresponded to a moderate level (5.0–15.0%) according to WHO threshold survey guidelines (Bennett et al., 2008). In contrast, a national research (Liu et al., 2023), which did not included INSTI-PDR, indicated that the overall prevalence of HIV-1 PDR showed an upward trend from 3.8% in 2016 to 6.9% in 2022 and a rapid increment in annual percent change of 11.17% were observed. Furthermore, the prevalence of HIV-1 PDR to NRTIs, PIs and NNRTIs showed the same epidemic pattern as the overall PDR in this study, respectively. To our knowledge, corona virus disease −19 started spreading in China at the end of 2019, and China government carried out large-scale prevention measures to control the spread of this novel virus throughout the whole country in 2020. We infer that prevention measures may disrupt the government’s free ART plans and make some patients temporarily interrupt their treatment programs, leading to a rapid increase of DR mutations. Resistant strains begin to spread out of original groups into other populations such as treatment-naïve MSM once prevention measures are lifted, and HIV-1 PDR will decline with the process of ART normalization.

Our founding indicated that the prevalence of INSTI-PDR presented an increase from 2.3% in 2018 to 2.7% in 2019, while a sharp decrease to 0.9% in 2021 was observed, and then increased significantly to 3.6% in 2022. To put these findings in a global context, the prevalence of INSTI-PDR reported in our study was lower than some countries, such as Canada (8.0%) (Hezhao et al., 2018) and Cameron (5.1%) (Benjamin et al., 2021), but higher than estimates from Uganda (1.2%) (Suzanne et al., 2021) and Korea (2.4%) (Sang-Min et al., 2023). The result of a domestic preliminary investigation showed that there was a low prevalence (0.8%) of INSTI-PDR in some provinces of China in 2018 (Song et al., 2021). Moreover, the prevalence of INSTI-PDR reported in our study was higher than some areas, such as Guangxi (3.1%) (Yan et al., 2023), Beijing (0.62%) (Yu et al., 2022), Jiangsu (1.7%) (Yin et al., 2021), Guangzhou (1.49%) (Lan et al., 2022) and Nanjing (2.5%) (Wang et al., 2023), but lower than estimates from China’s Yunnan (5.7%) (Deng et al., 2019), Taiwan (5.3%) (Chang et al., 2016) and Shenyang (3.7%) (Huang et al., 2019). And, all of INSTI mutation points were distributed in CRF01_AE and CRF07_BC, which significantly different from the distribution in subtypes A-G abroad (You et al., 2016). The prevalence of INSTI-PDR presented an obvious increase in this situation, suggesting that effects of INSTIs will face an unprecedented severe challenge in the future.

We found that a total of 48 transmission networks were circulating in the MSM population in Hebei, China. CRF01_AE was the most widespread subtype between cities, and CRF07_BC showed the strongest multi-city transmission. This study indicated that aged 25–49 years, cadre/staff/teacher, unmarried, minority, high school or below, initial CD4 counts >500 cells/μL, no STD history, homosexual partners ≥10, special survey, long-term infection, and drug resistance were associated with clustering in molecular transmission networks, and clustering frequency in this population was significantly related to CD4 counts, year and city. Furthermore, a substantial decrease was observed in clustering frequency from 37.5% in 2018 to 18.7% in 2022, with a concomitant increase in ART coverage from 78.7 to 91.1% over this period in Hebei, China.

However, 22.9% of 48 networks contained HIV-1 PDR mutations and 43.8% contained recently infected participants, and had a very strong transmission vitality. For example, five of seven major large networks (clusters 1–7) contained PDR mutations in this study, including two PI-mutation clusters, two INSTI-mutation clusters, one NNRTI and INSTI mutations clusters. And, recently HIV-1-infected individuals were circulating in four of five networks with PDR mutations. This suggests that CRF01_AE and CRF07_BC will be the most frequent HIV-1 strains in the MSM population in the future, and promote HIV epidemic of Hebei.

Compared with transmission links between Hebei and neighboring provinces proved by our previous reports (Lu et al., 2017b, 2020; Fan et al., 2022), our current work analyzed intracity and intercity transmission links of HIV-1 provincially. At the regional level, we found that the geographical distribution of clustering frequency was clustered in the central and northern part of Hebei, such as Qinhuangdao, Chengde, Baoding and Shijiazhuang. Six of seven large clusters with ≥5 participants were being in intercity links, with an apparent exception of cluster 3. The strongest intercity transmission for CRF07_BC was identified in Qinhuangdao-Baoding-Shijiazhuang-Cangzhou-Chengde-Tangshan. In cluster 3, strong intracity links for CRF07_BC were found in Qinhuangdao. The number of transmission networks identified in this study is not in accordance with the current level of HIV-1 epidemic in eleven cities of Hebei (Wang et al., 2021), which suggests that HIV-1 epidemic is growing rapidly in some cities with few HIV/AIDS cases, such as Qinhuangdao and Chengde. Although the success rate of ART indicated a significant increase from 95.0% in 2018 to 98.3% in 2022 according to our unpublished data, our findings show that HIV prevention and control will face severe challenges in the future.

Our current work has several limitations. First, the sample size in this study was limited because HIV/AIDS cases recently diagnosed were few at the HSPs in Hebei. Particularly, the precision of subtype distribution, PDR and network estimates in this area may be reduced due to the smaller sample size in some city such as Handan. Second, recent HIV-1 infection was tested only using HIV-1 LAg-Avidity, and recently HIV-1 infected MSM identified in our study were not confirmed again using the testing of HIV-1 viral load due to a fund shortage, which may be overestimate HIV-1 incidence. Lastly, only URFs were confirmed using NFLGs, however the most of HIV-1 subtype confirmation and the transmission network analysis were based on HIV-1 NFLPs, ignoring recombination within HIV-1 *gag* or *env* region. This may not reflect the actual HIV-1 subtype and intercity or intracity links of HIV-1 transmission. To minimize the above biases, longer gene fragments and more advanced methods to analyze HIV-1 subtypes, genovariation, and molecular transmission network are expected to improve the accuracy of study results later.

## Conclusion

Our study demonstrated that a total of 14 HIV-1 subtypes were circulating in the HSPs of Hebei province, reflecting HIV-1 epidemic in the MSM population is complex and severe. CRF01_AE, CRF07_BC, B and URFs were the four most predominant subtypes among MSM, and CRF07_BC and URFs indicated an increasing trend, respectively; however, CRF01_AE showed a decline trend. We analyzed HIV-1 PDR to PIs, NRTIs, NNRTIs and INSTIs, and found that the overall prevalence of HIV-1 PDR showed a substantial increase from 6.3% in 2018 to 7.9% in 2022. Furthermore, extensive HIV-1 strains bearing PDR were circulating in molecular transmission networks for major subtypes especially CRF01_AE and CRF07_BC. Therefore, it is urgent for us to implement more effective intervention measures to limit the further dissemination of HIV-1.

## Data availability statement

The original contributions presented in the study are publicly available. This data can be found here: partial HIV-1 NFLG sequences identified in this study have been deposited into GenBank with the accession numbers: ON007374, OR496590, OR400997, OP390172–OP390174, OP169133-OP169134, OK392124-OK392125, OQ513731-OQ513733, OP921950-OP921952, PP212966-PP212967.

## Ethics statement

The studies involving humans were approved by the Medical Ethics Committee of Hebei provincial center for disease control and prevention. The studies were conducted in accordance with the local legislation and institutional requirements. Written informed consent for participation in this study was provided by the participants' legal guardians/next of kin.

## Author contributions

XL: Conceptualization, Data curation, Formal analysis, Funding acquisition, Methodology, Resources, Software, Validation, Visualization, Writing – original draft, Writing – review & editing. YW: Data curation, Methodology, Resources, Writing – review & editing. LM: Data curation, Investigation, Resources, Writing – review & editing. ML: Software, Visualization, Writing – review & editing. YL: Data curation, Software, Writing – review & editing. NA: Data curation, Methodology, Writing – review & editing. XZ: Data curation, Methodology, Writing – review & editing. XT: Methodology, Writing – review & editing. QL: Conceptualization, Funding acquisition, Project administration, Resources, Writing – review & editing.

## Footnotes

^1^https://www.hiv.lanl.gov/components/sequence/HIV/crfdb/crfs.comp

^2^https://www.hiv.lanl.gov/content/sequence/BASIC_BLAST/basic_blast.html

^3^http://www.hiv.lanl.gov/content/index

^4^http://dbpartners.stanford.edu:8080/RegaSubtyping/stanford-hiv/typingtool/

^5^http://hivdb.stanford.edu/

## Glossary

AIDS: Acquired immune deficiency syndrome
HIV: Human immunodeficiency virus
DTG: Dolutegravir
ART: Antiretroviral therapy
DR: Drug resistance
PDR: Pretreatment drug resistance
PIs: Proteinase inhibitors
RTs: Reverse transcriptase inhibitors
INSTIs: Integrase strand transfer inhibitors
MSM: Men who have sex with man
URFs: Unique recombinant forms
CRFs: Circulating recombinant forms
ARDs: Anti-retroviral drugs
DRM: Drug resistance mutation
RTIs: Reverse transcriptase inhibitors
NRTIs: Nucleoside reverse transcriptase inhibitors
NNRTIs: Nonnucleoside reverse transcriptase inhibitors
NFLPs: Near full-length *pol* sequences
HSPs: HIV surveillance points
NFLGs: Near full-length genomes sequences
RT-PCR: Reverse transcription-polymerase chain reaction
WHO: World health organization
EVG: Elvitegravir
RAL: Raltegravir
BIC: Bictegravir
CAB: Cabotegravir
STD: Sexually transmitted disease
VCT: Voluntary consultation and testing.
